# Effectiveness of passive ultrasonic irrigation in improving elimination of smear layer and opening dentinal tubules

**DOI:** 10.4317/jced.51297

**Published:** 2014-02-01

**Authors:** Sandra Mozo, Carmen Llena, Nicholetta Chieffi, Leopoldo Forner, Marco Ferrari

**Affiliations:** 1 DDS. Private practice in Endodontics; 2 MD, DDS, PhD. Professor. Department of Stomatology. University of Valencia. Spain; 3 DDS, PhD. Associate profesor. Department of Stomatological Sciences. University of Siena. Italy; 4 MD, DDS, PhD. Professor. Department of Stomatological Sciences. University of Siena. Italy

## Abstract

Objective: To compare the ability to eliminate debris and open dentinal tubules of different ultrasound irrigation procedures. Study Design: Forty extracted single-rooted human teeth were instrumented with mechanical rotatory instrumentation, and divided into four groups: (n=10) according to the final irrigation technique: group A (control), 2.5% NaOCl irrigation with the Miraject needle and no ultrasonic agitation; group B, passive ultrasonic irrigation (PUI) with 2.5% NaOCl and Irrisafe 20 tips; group C, PUI with 2.5% NaOCl and Irrisafe 25 tips; group D, PUI with 2.5% NaOCl and K 25 tips. The amount of debris and the number of opened dentinal tubules was established by scanning electronic microscope. Data were compared using the Kruskal Wallis test. Results: Irrisafe tips (groups B and C) opened up more dentinal tubules and eliminated more debris than conventional irrigation (p<0.05) in the apical third. The middle third shows no significant differences between groups. Irrisafe 25 was more effective than conventional irrigation and K tips (p<0.05) in the coronal third. Conclusions: Ultrasonic activation of the irrigation with Irrisafe tips was the most effective procedure for eliminating the debris and opening up dentinal tubules, especially in the apical third.

** Key words:**Pasive ultrasonic irrigation, irrigation, sodium hypochlorite.

## Introduction

Irrigation solutions act mainly as lubricants and as cleaning agents during biomechanical endodontic treatment, improving permeability of the canal throughout its length and the elimination of the contaminated dentin (1,2). To ensure effective action, irrigants must be in direct contact with the entire canal walls, especially in the apical third. Presence of vapor lock in this portion might also hinder the exchange of irrigants and affect the debridement efficacy of irrigants (1).

Different techniques have been proposed to improve irrigant distribution within the root canal system (3,4). Passive ultrasonic irrigation (PUI) is a noncutting irrigation protocol applied with ultrasonically activated files, and could be used with a continuous or intermittent flow of irrigant (5). For intermittent flush the irrigant is injected with a syringe that is filled several times after each cycle of ultrasonic activation. The amount of irrigant that is flushed through the apical region of the canal can be controlled by the depth of penetration of the syringe and the volume of irrigation administered. This control is not possible when continuous flow is used. Both flush methods have proved to be equally effective in removing dentin residue from root canal when used for three minutes (5,6).

In PUI technique, energy is transmitted from a file or smooth oscillating wire to the irrigation by ultrasonic waves, producing a stream and cavitation of the irrigation solution disrupting the vapor lock (7,8). Transient cavitation only occurs when the file can vibrate freely in the canal or when the file touches lightly the canal wall. When the root canal has already been shaped, the file or wire can move freely and the irrigant can penetrate more easily into the apical part of the root canal system and the cleaning effect will be more powerful (9,10). Using this noncutting methodology, the potential to create aberrant shapes within the root canal can be reduced to a minimum (10)

A file larger than size 15 or 20 will only oscillate freely in a wide root canal, a size 25 file may in fact produce less acoustic streaming than a size 15 and 20 file, consequently, using a file larger than 20 may be considered fundamentally different from the basic principle of PUI (11). The surface property of the file is important for the enhancement of cavitation, a smooth file with sharp edges and a square cross-section produced significantly more transient cavitation than a normal K-file (12,13).

The use of sodium hypochlorite (NaOCI) as final irrigant combined with PUI has been shown to be more effective that syringe needle irrigation in removing debris, bacterial reduction and smear layer removal (14,15).

The aim of this study is to compare the ability in cleaning root canal walls of conventional syringe versus intermittent PUI using K files, and Irrisafe 20 or 25 tips in the final irrigating procedure.The null hypothesis is that there are no differences in canal wall cleaning after PUI irrigation with Irrisafe tips and K tips and there are no differences between ultrasonic irrigation and conventional irrigation.

## Material and Methods

Forty single-rooted human premolars extracted for periodontal reasons were used for this study. The teeth were placed in 2.5% NaOCl solution for 5 min and then in saline solution. All the teeth were radiographed (70 kV–0.08 s) to verify the presence of single canal, mature apex, absence of any resorption or endodontic treatment and a lower curvature than 5 degrees evaluated by the Schneider technique (16).

Crowns were sectioned at the cemento-enamel junction. The working length was measured by deducting 1 mm from the length recorded when tips of K-flexofiles 15 (Dentsply Meallefer Tulsa, OK, USA) were visible at the apical foramen. All the canals were prepared by the same operator with the MTwo rotary system (VDW, Munich, Germany) following basic sequence (10/.04, 15/.05, 20/.06, 25/.06) and file 30/.05, at 300 rpm. Between files, irrigation with 2.5 ml of 2.5% NaOCI was administered with a 27G Miraject needle (Hager Werken, Duisburg-Grobenbaum, Germany). The total volume of NaOCl irrigant used during instrumentation was 12.5 ml. The insertion depth of the irrigation needle was 1mm less than the working length. Then, all the canals were irrigated with 2 ml of 17% liquid EDTA (Acteon Pharma, Merignac, France) for 2 minutes. The exterior part of the apical third of each root was covered with a composite resin to prevent irrigant from dripping through the apical foramen. This was done after placing a calibrated gutta-percha cone at the working length in order to avoid composite intrusion into the apex. The cone was removed after resin curing.

Teeth were randomly divided into four groups (n=10) according to the final irrigation technique: group A (control), irrigation with 2.5% NaOCl with the Miraject needle and no ultrasonic agitation; group B, PUI with 2.5% NaOCl and Irrisafe 20 tips; group C, PUI with 2.5% NaOCl and Irrisafe 25 tips; group D, PUI with 2.5% NaOCl and K 25 tips.

Irrisafe ultrasonic tip is a stainless steel 20/.00 or 25/.00 one (Acteon, Merignac, France). Irrisafe and K25 tips, were activated through a 5.5W 30kHz piezoelectric ultrasound unit Suprasson P5 Booster (Satelec Acteon, Merignac, France).

Groups B, C and D were irrigated with 5 ml of 2.5% NaOCI followed by the passive irrigation technique with intermittent flush which consisted in applying 3 cycles of ultrasonic activation of the irrigant for 20 seconds each so that each canal was subjected to 1 minute of passive ultrasonic irrigation. Irrigation with 2 ml of NaOCI was carried out between cycles. The ultrasonic tip was placed coronally 1 mm to the working length, the file was kept centered in the canal and 2-3 mm apical-coronal movements were made (8,12), the amount of final irrigating NaOCl solution was 11 ml.

**Figure 1:**
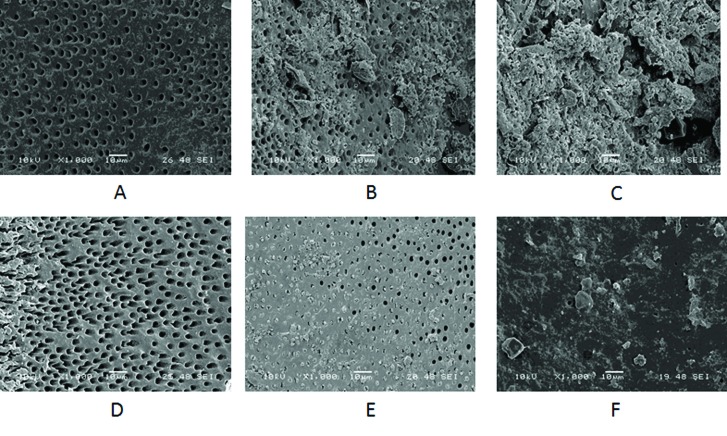
A) 0 = no debris; B) 1 = some debris of <20 μm; C) 2 = a lot of debris of >20 μm); D) 0 = all open; E) 1 = some open; F) 2 = all closed.

After final irrigation the roots were fractured in the buccolingual direction using a chisel and a mallet. For each specimen, the half containing the most visible part of the apex was conserved and coded. These specimens were mounted on metallic stubs, coated with palladium-gold, and then examined under a scanning electron microscope (SEM) (Geol JSM-6060VL, Westmont, USA) under 1000x and 10 KN and 16 WD. The whole area of the root canal wall was evaluated at 10, 6 and 2 mm from the coronal limit (apical, middle and coronal third, respectively) in order to establish the amount of debris according to the criteria used by Serafino et al. in 2006 (17): 0= no debris, 1= little debris of < 20 µm, 2 = a lot of debris >20µm (Fig. 1). In the same area, the amount of opened dentinal tubules were also evaluated according to the following criteria: 0= all opened, 1 = some opened, 2 = all closed (Fig. 1). The evaluation and scoring were conducted by 2 independent evaluators in a blinded manner. The scores were compared and, when a difference was found, the evaluators jointly examined the sample. If they could not reach agreement, a third evaluator helped with scoring the sample. Inter-reliability was established with Cohen´s Kappa test (18).

Data were analysed with SPSS 17.0 statistical software. Kruskal-Wallis test was used to compare between final irrigation techniques. Mann-Whitney U test was used for pairwise comparisons between techniques. Friedman test was used for comparisons among root canal thirds. Wilcoxon signed rank test was used for pairwise comparisons among root canal thirds. The significance level was set at p<0.05.

## Results

There was excellent inter-observer agreement for the debris and opened tubules scores (0.92 and 0.89 respectively).

-Debris elimination

Final irrigation with conventional syringe (group A), eliminates all or the majority of debris in 63% of the samples in all thirds, as can be seen in  Table 1. PUI applied with Irrisafe tips (groups B and C) and PUI applied with K tips in 93% and 80% of samples, respectively. Significant differences were obtained between conventional irrigation and PUI applied with Irrisafe tips (p<0.05).

**Table 1:**
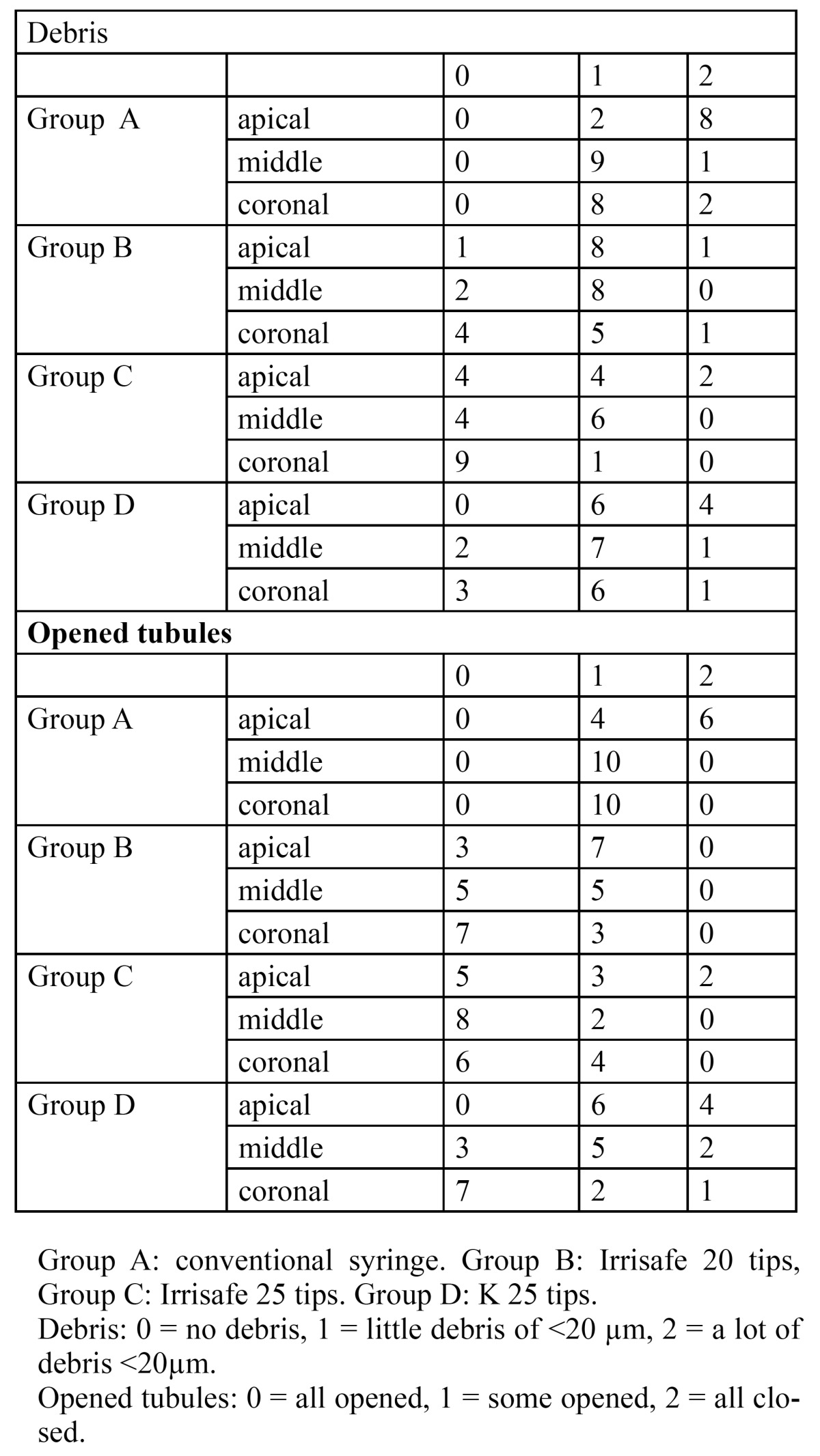
Debris and opened dentinal tubules scores at the apical, middle and coronal thirds in the different groups.

In the apical third, Irrisafe tips (groups B and C) eliminated more debris than the conventional irrigation ( Table 2); in the middle third, the lowest score of debris was obtained in the group C without differences between techniques; and in the coronal third, PUI with Irrisafe 25 tips (group C) was more effective than irrigation with a conventional syringe (group A) or K tips (group D).

**Table 2:**
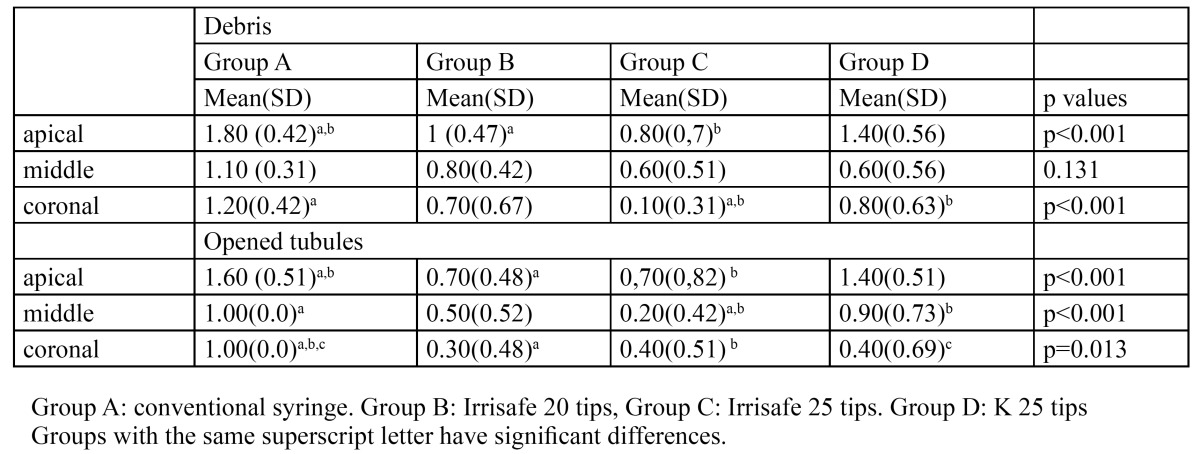
Mean and standard deviation (SD) values of debris and opened tubules for each group and third.

- Opened tubules

All or almost all tubules were opened in the 80% of group A, 100% in the group B, 93 % in the group C and 90 % in the group D ( Table 1), significant differences were obtained between conventional irrigation or PUI applied with K tips and Irrisafe tips (p<0.05).

In the apical third, groups B and C opened significantly more tubules than group A ( Table 2), without differences between them; in the middle third, group C opened significantly more tubules than groups A and D; and in the coronal third, significant less amount of tubules were opened in the group A.

## Discussion

This study rejects the null hypothesis that the irrigation technique with conventional syringe is equally as efficient as irrigation techniques using intermittent PUI with different instruments. The null hypothesis that intermittent PUI irrigation with Irrisafe tips and K tips is equally as efficient in cleaning root canal walls was also rejected.

Instruments must be able to move freely in the canal during ultrasound irrigation, as contact of the instrument with the walls would limit the acoustic microstream effect, thereby reducing the flush of irrigation throughout the canal system and reducing the effectiveness of cleaning and disinfection (11). This indicates that a certain amount of root canal enlargement is necessary to allow sufficient irrigation. Huang et al. (19) reported that a larger apical size allowed better apical flushing by the irrigant and that a larger taper provided better irrigant exchange between apical and coronal root canal areas. Khademi et al. (20) showed that an apical instrumentation to a size 30 file with 0.06 coronal taper is effective for penetration of irrigants to the apical third. In this study all samples were shaped with a final 30/0.05 file” over a previous shaping with a 25/0.96 file.

There is a general consensus that PUI is more effective than conventional syringe and needle irrigation at eliminating debris (1,21,22). According our findings, PUI eliminates more dentin debris than conventional irrigation at all evaluated root-levels. Although PUI showed to be better than conventional needle irrigation according to a previous study, debris could not be completely eliminated using PUI with 1% NaOCl during 10 seconds (23). Other studies (4,13) reported that EDTA or an EDTA and NaOCl combination did not completely eliminate the debris of the apical walls after its ultrasonic activation. In our study ultrasonic irrigation, independently of the instrument (Irrisafe 20 or 25 or K file) was more effective in eliminating debris and opened tubules than conventional irrigation, but in any group achieved complete opening of the tubules or complete removal of debris.

In this in vitro experiment, the majority of the remaining debris were located in the apical third. The same is reported by the great majority of authors (12,24,25). When Irrisafe 20/0.00 tips were used, only in one sample, dentin debris larger than 20µm were found, also in this group all or almost all dentinal tubules were opened ( Table 1).

PUI with Irrisafe tips and an intermittent flush technique of three 20-second applications is just as effective in eliminating debris as laser activated techniques (26), however one single 20-second application with Irrisafe tips and ultrasound was significantly less effective, then, the time the irrigation remains in the canal is a factor to be taken into account during PUI (27). Although none of the techniques described achieves to eliminate the debris completely throughout the canal (25,27,28,29), in this study, intermittent PUI for three 20-second applications and 5.5W of intensity cycles with Irrisafe tips proved to be more effective than conventional irrigation.

Although some authors report that K-files or parallel-shaped and noncutting ultrasonic instrument can be equally effective for PUI application (30), K 25 files appeared to be less effective eliminating smear layer and opening tubules tan Irrisafe tips. Although there were not significant differences in the apical third, there were a bigger amount of debris and a less number of opened tubules with K files when compared with the Irrisafe tips.

It can be concluded that irrigation with conventional syringe in the initial preparation stage followed by a final phase of passive ultrasound irrigation (PUI) with intermittent flush and using Irrisafe tips is effective for cleaning root canals.
